# A model for extra-axonal diffusion spectra with frequency-dependent restriction

**DOI:** 10.1002/mrm.25363

**Published:** 2014-07-15

**Authors:** Wilfred W Lam, Saâd Jbabdi, Karla L Miller

**Affiliations:** Centre for Functional MRI of the Brain, University of OxfordOxford, UK.

**Keywords:** diffusion MRI, diffusion spectrum, extra-axonal space, extracellular space, restricted diffusion, hindered diffusion

## Abstract

**Purpose:**

In the brain, there is growing interest in using the temporal diffusion spectrum to characterize axonal geometry in white matter because of the potential to be more sensitive to small pores compared to conventional time-dependent diffusion. However, analytical expressions for the diffusion spectrum of particles have only been derived for simple, restricting geometries such as cylinders, which are often used as a model for intra-axonal diffusion. The extra-axonal space is more complex, but the diffusion spectrum has largely not been modeled. We propose a model for the extra-axonal space, which can be used for interpretation of experimental data.

**Theory and Methods:**

An empirical model describing the extra-axonal space diffusion spectrum was compared with simulated spectra. Spectra were simulated using Monte Carlo methods for idealized, regularly and randomly packed axons over a wide range of packing densities and spatial scales. The model parameters are related to the microstructural properties of tortuosity, axonal radius, and separation for regularly packed axons and pore size for randomly packed axons.

**Results:**

Forward model predictions closely matched simulations. The model fitted the simulated spectra well and accurately estimated microstructural properties.

**Conclusions:**

This simple model provides expressions that relate the diffusion spectrum to biologically relevant microstructural properties. Magn Reson Med 73:2306–2320, 2015. © 2014 The authors. Magnetic Resonance in Medicine Published by Wiley Periodicals, Inc. on behalf of International Society of Medicine in Resonance.

## INTRODUCTION

Diffusion-weighted magnetic resonance imaging has enormous potential to noninvasively estimate the geometric properties of biological tissues. This remarkable capability is grounded in the sensitivity of the natural motion of water molecules to their microenvironment, which imparts a characteristic change to water diffusion. This altered diffusion provides a signature of the size and shape of the compartments to which it is confined 1–3. For example, in the brain, the diameter of axons in white matter alters the apparent diffusion coefficient of water depending on the time that molecules are allowed to explore the intra-axonal space 4–10. With sufficiently accurate models for how this behavior translates into a detectable change in the diffusion-weighted magnetic resonance imaging signal, one can infer geometric properties of the tissue microstructure. In the context of biological tissues, many of these geometric properties relate to tissue function and health 11, making such a technique of great interest as a biomarker.

An alternative to this traditional approach of measuring at a range of diffusion times is to consider the translational motion of the diffusing molecules. This so-called diffusion spectrum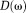 decomposes molecular movement into a range of temporal frequencies (*ω*), resulting in a frequency-dependent diffusion coefficient. The diffusion spectrum should not be confused with the unrelated technique of diffusion spectrum imaging, which aims to estimate the diffusion propagator. Formally,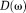 is defined as the Fourier transform of the velocity autocorrelation function of diffusing particles 12. The velocity autocorrelation function describes how long particles continue in their current direction until they change direction or speed, either due to scattering against another particle or encountering a barrier. For a given particle, its velocity over time can be expressed in terms of a weighted sum of velocity at different temporal frequencies (i.e., a Fourier transform of the velocity time course). The diffusion spectrum is the equivalent decomposition of the expected (ensemble average) velocity autocorrelation. Equivalently, the diffusion spectrum is the velocity power spectrum of an ensemble of diffusing particles. This diffusion “power spectrum” relates to the geometry of the environment to which molecules are confined, and thus geometric properties of the environment can be estimated from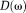, given an appropriate model.

Measurements of the diffusion spectrum require the use of oscillating gradients, rather than the more conventional pair of gradients pulses 13. These oscillating gradients cause a given water molecule to accrue a large positive (or negative) phase offset if a significant component of motion of the molecule along the direction of the gradient is positively (or negatively) correlated with the gradient waveform. Molecular motion at other frequencies will be orthogonal to the gradient waveform, resulting in negligible phase offsets and making the MR signal insensitive to this motion. An ensemble of molecules, each with random phase offset at a given frequency, will thus accrue a distribution of phase offsets. This will, in general, lead to signal loss (similar to conventional diffusion weighting) and, under some circumstances, a coherent phase offset 14–16. Oscillating gradient measurements have been demonstrated for both in vivo 17–20 and ex vivo 21,22 brain tissue. Most in vivo measurements have been made on preclinical scanners due to the difficulty of achieving sufficient diffusion weighting using clinical scanners 23.

Microstructural characterization of tissue typically begins by assuming some simple geometry for which the diffusion spectrum has been derived and relating this to the diffusion-weighted magnetic resonance imaging signal in an oscillating gradient experiment. In the brain, cylinders are often used to model axons 24–26. This is typically combined with a second compartment intended to account for the extra-axonal space (EAS). The diffusion spectrum of the EAS is known not to be spectrally flat 27,28 owing to the tortuous nature of the EAS 29. This has also been observed in nonoscillating gradient experiments 30. However, in certain models, the EAS diffusion spectrum has been approximated as a constant over all frequencies, implying that “hindered” diffusion in the EAS is spectrally equivalent to free diffusion, but with a lower diffusion coefficient (i.e., diffusion is slow, but the spectrum has no shape) 31. This approximation is valid at low frequencies, but will likely lead to biases in fitting geometric parameters such as restricted volume fraction or axon diameter at higher frequencies, as will be demonstrated here through simulations.

The goal of the current work is to propose a model for the diffusion spectrum in the EAS with improved accuracy that may yield white matter microstructural information. Models of the intra-axonal space typically assume that water molecules strictly remain inside axonal cylinders over the time scale of a measurement (“restricted” diffusion). By comparison, an EAS model must describe a more complicated geometry in which water molecules will tend to remain in the confining space between axons where they are at the beginning of a measurement, but will occasionally diffuse outside to an adjacent space. Closed-form solutions are unlikely to describe such complicated geometries, particularly biological systems with a distribution of confining space sizes and random packing. Instead, the proposed EAS model aims to retain much of the simplicity of previous work by considering the confining spaces between axons to be approximated by cylinders, while encapsulating the ability of molecules to diffuse between these spaces with a component that is spectrally flat. This hybrid approach can be considered as a kind of exchange model in which water molecules move between regimes of restriction (when confined to an extra-axonal “cylinder”) and hindrance (when diffusing between cylinders) and can be compared to the work of Callaghan et al. 3 and Kuchel et al. 32. Versions of the model are presented for both periodically packed, uniform cylinders and randomly packed cylinders with a distribution of radii. The accuracy of these models is assessed through comparison with Monte Carlo simulations of these geometries at a range of spatial scales, demonstrating good agreement despite the simplified nature of this model.

## THEORY

In this section, we present a conceptual framework for the diffusion spectrum in the EAS. We begin with a description of the properties of the diffusion spectrum and then present simulations of diffusion in the EAS. Next, we build on this to present a new model for the EAS that aims to strike a balance between simplicity and explanatory power.

### The Diffusion Spectrum

The diffusion spectrum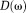 is the Fourier transform of the particles' average velocity autocorrelation; that is, the power spectrum of particle velocities. In the absence of barriers, diffusion is “free” and is driven simply by the scattering of molecules with each other. The velocity autocorrelation for free diffusion is effectively instantaneous decorrelation (a delta function), which Fourier transforms to a constant diffusion spectrum.

This default shape of the diffusion spectrum is altered by the presence of any barriers that impede movement. This general property holds for both confining spaces from which particles cannot escape (restricted diffusion) and porous microstructure with communicating spaces (hindered diffusion). The primary difference between restriction and hindrance is that the latter enables molecules to displace an arbitrary distance given sufficient time, although displacement rates are slower than for free diffusion.

As we will see, confining spaces (whether restrictive or hindering) correspond to a reduction of the diffusion coefficient at low frequencies, while high frequencies are less affected. In restricting geometries, particles must have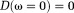 in order for particles to remain inside the confining space; for hindering geometries, the diffusion spectrum is reduced, but nonzero, at zero frequency. Reduced diffusion under hindrance is characterized by the microstructural “tortuosity” 27, defined by the ratio of displacements for hindered and free diffusion at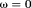 33. Tortuosity has been derived analytically for certain regular geometries 34,35, but is more difficult to calculate for arbitrary geometries.

This leads to the general property that, in the presence of restricting or hindering barriers, the diffusion spectrum transitions from free diffusion behavior at high frequencies (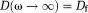, the free diffusion coefficient) to reduced diffusion at low frequencies. The transition from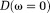 to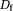 is well-studied for pure restriction, resulting in a known relationship between the length scale of the restrictions and the specific shape of the diffusion spectrum 12,31. In contrast, the shape of the diffusion spectrum has received less attention for hindered geometries, which are more complicated and often less suited to closed-form calculations.

### Preliminary Simulations

**Figure 1:**
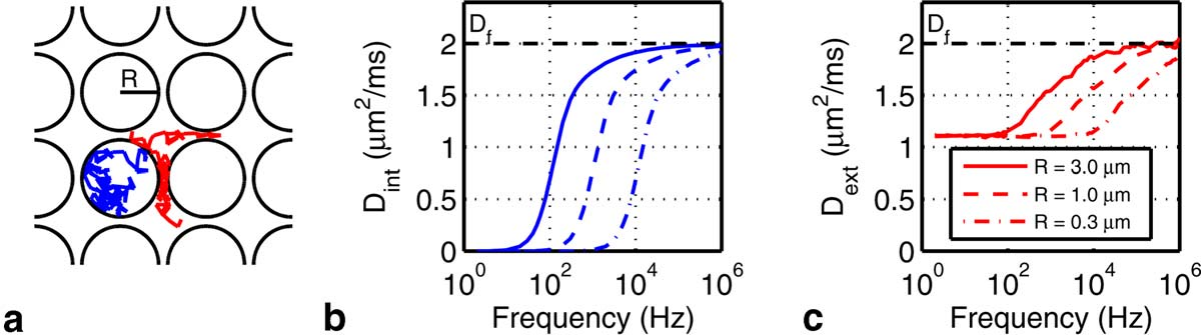
a: Square packed cylinders (axial view) of radius *R* with representative paths of diffusing intra- (blue) and extracylinder (red) particles. The corresponding diffusion spectra for the (b) intra- and (c) extracylinder spaces for three different cylinder radii (with a cylinder center-to-center separation to diameter ratio of 1.12).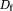 is the free diffusion coefficient.

Throughout this work, Monte Carlo simulations are used as a ground truth to establish the properties of the diffusion spectrum of water in the space surrounding different geometric configurations of cylinders representing the EAS. We present simulated geometries that range from regular lattices of cylinders with uniform radii to random packings with a distribution of radii. The full details of these can be found in the Monte Carlo simulations subsection. In this section, we consider a preliminary set of simulations of the EAS to demonstrate the characteristic features of this space and motivate the EAS model that follows. The diffusion spectra for the intra- and extracylinder spaces of a simple square packing geometry are shown in Figure 1.

The spectrum for the interior of an impermeable (restrictive) cylinder approaches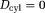 at low frequency and asymptotes to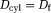 at high frequency with a sigmoidal transition between these two regimes 12. This behavior directly follows from the fact that a constant or slowly varying velocity (low frequency) results in a large net displacement. Restrictive spaces like impermeable cylinders thus place limitations on velocity at low frequencies; in particular,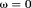 corresponds to mean velocity autocorrelation, and any nonzero mean velocity autocorrelation would eventually cause a particle to leave the compartment. Restrictive compartments are thus characterized by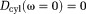 and, more generally, a damping of mean velocity at low frequencies.

Unlike restricted geometries, molecules in hindered spaces like the EAS can displace an arbitrary distance given sufficient time. Based on this observation, EAS molecules have been argued to behave like a slower version of free diffusion with a nonzero diffusion coefficient at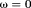 (i.e., some average velocity). Recall that the mean squared free diffusion displacement *x* in one dimension is described by Einstein's equation 36

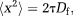
1where *τ* is the diffusion time of the measurement. The ratio of free to hindered mean diffusion displacements 33 is referred to as “tortuosity” (denoted *λ*):

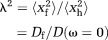
2

Analytical expressions for the tortuosity of the space around periodically packed cylinders are given in 34,35, and one can then rearrange the equations above to write

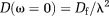
3

This property holds at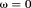 by definition (since a net displacement is associated with zero frequency). Although the diffusion spectrum of the EAS is known not to be spectrally flat 27,28, some previous models 31 have further assumed that this value holds at all frequencies resulting in a flat spectrum that is similar to free diffusion, but with a reduced diffusion coefficient

Interestingly, our basic Monte Carlo simulations (described in detail below) demonstrate that the EAS spectrum has a shape similar to the spectrum for an impermeable cylinder (intra-axonal). While diffusion for restrictive and hindering geometries deviates at low frequencies, they are observed to be convergent at high frequency. Diffusion at high frequency has been related to the surface-to-volume ratio (*S*/*V*) of the environment 37. This property is similar to that of short diffusion time, pulsed gradient measurements: for short diffusion time (or high oscillation frequencies), only molecules a very short distance from a reflecting surface experience altered diffusion behavior, while all other molecules in the volume experience free diffusion.

The diffusion spectrum for a hindering extracylinder space, which is a compelling model for extra-axonal microstructure, to date, remains impartially described. Previous models of the EAS spectrum are accurate for low 27 and high 37 frequency asymptotes. The transition between these extremes is the subject of the present work, which aims to present a simple model that approximates the entire spectrum.

### Basic Exchange Model

The observed shape of the EAS spectrum shown in Figure 1c is perhaps not surprising given what we know of the space between cylinders. For cylinders that are completely abutting, the EAS water is trapped in restrictive “pores,” much like the water inside impermeable cylinders. As we loosen the cylindrical packing (reduce tortuosity), gaps appear through which water can communicate between pores. EAS water will still tend to remain trapped in the pores, but will also occasionally diffuse between them. Thus, depending on the packing (tortuosity), water molecules can be considered to spend a variable fraction of their time behaving as restricted (trapped in pores) or free (moving between pores).

This description is at the heart of our proposed model, which expresses the EAS diffusion spectrum as a combination of free and restricted diffusion:

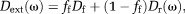
4where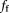 represents the fraction of the time a given particle is “free” (moving between pores), while for the remainder of time the particle is “restricted” to a pore. The model assumes that, as molecules migrate between pores, during the migration period they are characterized by a flat spectrum and at all other times by a restricted spectrum. We can thus consider this to be a type of exchange model, in which a given molecule exchanges between different regimes of behavior (rather than physical compartments). For this to be true, there must be fast exchange between the two regimes. Order of magnitude calculations (not shown) indicate the measurement is in the fast exchange limit described by Lee and Springer 38. Also simulations at *b* = 0.5, 1, and 2 ms/µm^2^ for regular and random packing yield the same diffusion spectrum.

The free diffusion spectrum is frequency independent and simply given by the diffusion coefficient of free water (i.e., it is identical to the spectrum for free diffusion). Given that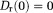, we can combine Eqs. 2 and 4 to determine that

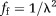
5

This states that the more tortuous the EAS (large *λ*), the less time molecules spend freely diffusing between pores.

**Figure 2:**
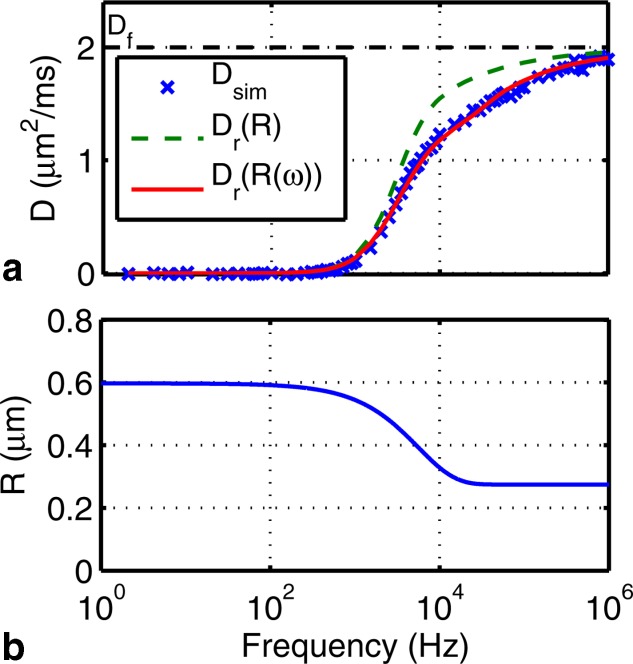
a: The “restricted component” of a simulated EAS spectrum (×) calculated by subtracting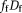 from the EAS spectrum of square packed cylinders. Overlaid are the diffusion spectra of a cylinder of radius *R* (– –) with the same volume as the EAS and that of the proposed variable radius model (–). b: The variable radius corresponding to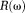 in a.

The form that the restricted component should take is less obvious given the complicated geometry of the EAS (i.e., the space between cylinders shown in Fig. 1a). Figure 2a isolates the restricted component by subtracting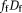 from a simulated EAS spectrum. It is clear that the shape of the restricted spectrum is similar to that of an impermeable cylinder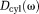 12 given in Appendix A and, in fact, comparing the spectrum of the EAS to that of a cylinder with the same total volume as the EAS gives a surprisingly reasonable prediction (dashed green line) of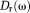.

However, this simple model for the restricted component (a cylinder of matched volume) does deviate significantly from the simulated spectrum in the transition region. Were we to use this model, we would expect a significant bias in any fit to data. Comparing simulations to restricted cylinders at a range of radii (not shown), it is clear that no radius captures the shape of the restricted component of the EAS spectrum. In particular, the EAS spectrum approaches the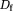 asymptote more slowly than the spectrum for a restricted cylinder. This difference is likely due to the deviation of the shape of the EAS from a cylinder. While one might hope to derive a complicated expression for the geometry external to abutting cylinders, such a model would further need to capture the change in geometry with looser packing to be useful. We instead take the approach of extending the cylinder model, which has the benefit of both simplicity and similarity to the EAS.

### Improved Exchange Model with Variable Radius

The agreement of the model with the simulated spectrum can be improved by allowing the radius of the cylinder to vary with frequency according to

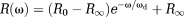
6

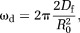
7in which *R*_0_ and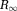 are the radii at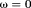 and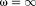, respectively, and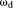 describes an exponential rate of decay between the two values. The expression for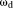 is adapted from the equation for mean diffusion displacement (Eq. 1). That is,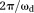 corresponds to the diffusion time, which is the time required for one standard deviation of diffusing particles to displace by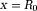. At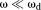, particles have diffusion displacements consistent with those within a cylinder of radius *R*_0_. At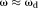, particles have displacements consistent with those within a cylinder with a radius between *R*_0_ and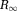.

The improvement in model agreement is demonstrated in Figure 2a (solid line), suggesting that the EAS can be described by a restrictive component with an effective radius that depends on frequency. The dependence of *R* on frequency is plotted in Figure 2b.

To calculate the EAS diffusion spectrum for randomly packed cylinders, we consider *R*_0_ to be a distribution and perform a weighted sum of the diffusion attenuation of each pore:

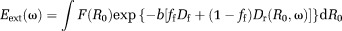
8where *F* is the distribution of *R*_0_, weighted by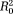, and normalized by its sum and *b* is the diffusion weighting. Note again that completely parallel cylinders are assumed. In the case of any orientation distribution, this equation will need to be modified to account for the projections along the gradient direction. We can then convert to an effective diffusion spectrum for all the pores:

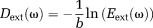
9

As will be demonstrated in the Results section, Eqs. [4]–[9] closely match the simulated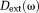 for regularly and randomly packed cylinders.

### Interpretation of Model

For square and hexagonally packed uniform cylinders and randomly packed cylinders with a gamma distribution of radii, we found the following relations to agree with simulated spectra across a wide range of cylinder sizes and packing densities (as will be shown in the Results section).

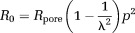
10

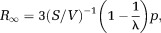
11where the effective pore radius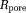 is the mean distance between the center of the pore and the walls of the cylinders and *p* is the fractional separation between adjacent cylinders. We define the fractional separation as

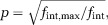
12where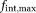 is the maximum cylinder volume fraction for a set of cylinders with a given radius distribution and packing type (e.g., square, hexagonal, and random) and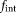 is the cylinder volume fraction. *p* = 1 for abutting cylinders.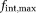 is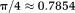 for square packed uniform cylinders;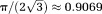 for hexagonally packed uniform cylinders; and can be determined numerically for randomly packed cylinders with a distribution of radii (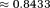 for the distribution used in this work). For the two cases of regular packing, *p* is also equivalent to the ratio of cylinder center-to-center separation to cylinder diameter, which we denote as *L* and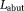, respectively.

**Figure 3:**
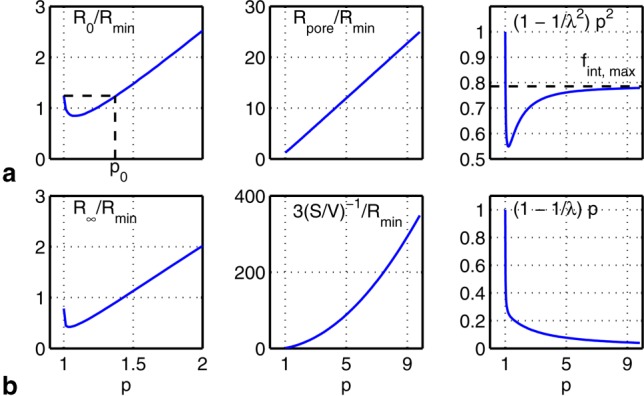
Plots of the model parameters (a) *R*_0_ and (b)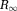 and their subfactors (normalized to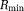, an equivalent radius for the EAS area under the tightest possible packing) as a function of fractional separation *p* for square packed cylinders. The value of *p* (denoted *p*_0_), when *R*_0_ for abutting and nonabutting cylinders are equal, and the abutting cylinder volume fraction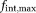 are indicated. The curves for hexagonal packing (not shown) are similar.

The behavior of the model parameters and their subfactors as a function of *p* is plotted in Figure 3. The symbols used in the model are summarized in Table1. The remainder of this section gives a qualitative explanation of Eqs. 10 and 11.

**Table 1:** List of Symbols in the EAS Diffusion Spectrum Model

Symbol	Description
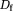	Free diffusion coefficient
*λ*	EAS tortuosity (Eq. [2])
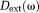	EAS diffusion spectrum (Eq. [4])
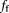	Fraction of EAS particles behaving as if freely diffusing (Eq. [5])
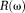	Radius of restricted diffusion component (Eq. [6])
*R*_0_	Radius of restricted diffusion component at 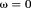 (Eq. [10])
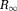	Radius of restricted diffusion component as 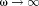 (Eq. [11])
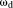	Transition rate from *R*_0_ to 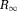 (Eq. [7])
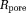	Effective pore radius
*S*/*V*	Ratio of pore surface area to volume
*P*	Fractional cylinder separation
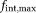	Cylinder volume fraction under tightest possible packing
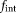	Cylinder volume fraction

The *R*_0_ parameter represents the effective radius of the restricted component when particles have had sufficient time to thoroughly sample the EAS (at low frequencies). When the cylinders are abutting,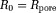. When the cylinders are slightly nonabutting,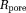 overestimates *R*_0_ because particles within the narrow “channels” between cylinders experience a more restricted space. However, as the gap between adjacent cylinders becomes greater, these channels decrease in significance and the value of *R*_0_ becomes increasingly driven by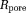. This transition occurs at the value of *p* where *R*_0_ for nonabutting and abutting cylinders are equal, which we denote as *p*_0_. Indeed, for square and hexagonally packed cylinders, *p*_0_ corresponds within 6% to the packing density at which the gap between adjacent cylinders is equal to the pore radius for abutting cylinders (i.e.,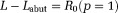; noted as *p*_0_ in Fig. 3a). For randomly packed cylinders,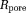 varies with each pore leading to a distribution of *R*_0_, but these general principles otherwise hold.

The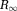 parameter represents the effective radius of the restricted component at high frequencies, reflecting short time periods during which particles only sample the EAS close to their initial position. This overall pattern is indicated in Figure 3b.

We would like to emphasize that the combination of our improved model with frequency-dependent radius, on the one hand, and Eqs. [10]–[12], on the other, serve two purposes. The frequency dependence allows us to have a good fit to the data at all frequencies. The empirically derived relationships described in Eqs. [10]–[12] give a description for this frequency dependence that is valid over a wide range (three orders of magnitude) of pore sizes, as will be clear in the Results section.

## METHODS

### Monte Carlo Simulations

**Figure 4:**
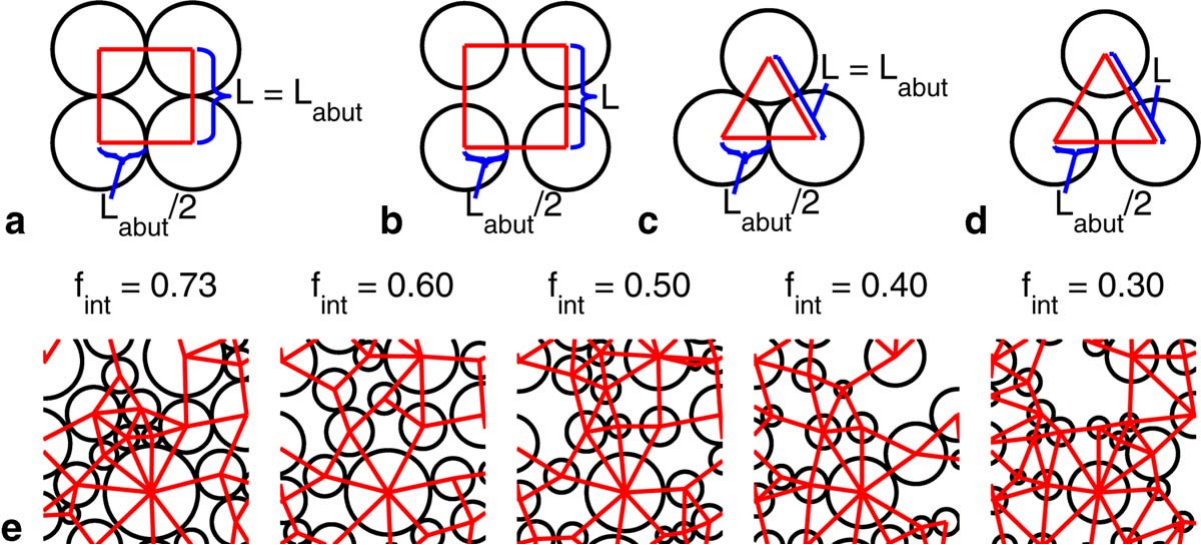
Axial view of (a) abutting and (b) nonabutting square packed cylinders with center-to-center separation *L* and abutting center-to-center separation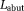 labeled. c, d: As a and b, but for hexagonally packed cylinders. e: Axial view of randomly packed cylinders with gamma-distributed radii and various cylinder volume fractions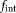 (only 4% of each tile of cylinders is shown). Pore boundaries are indicated by straight lines.

This model was applied to the periodically and randomly packed cylinders illustrated in Figure 4. Periodically packed cylinders were chosen for ease of relating model parameters to the cylinder geometry (packing type, cylinder radius, and cylinder separation). Randomly packed cylinders were chosen to approximate axon bundles and test the applicability of this model with more realistic but complex geometries.

We conducted extensive Monte Carlo simulations to define the ground truth for diffusion spectra over a range of geometries surrounding different packings of cylinders. MR signal attenuation was simulated in Camino 39,40 for the geometries depicted in Figure 4.

For square and hexagonally packed cylinders, the various environments were explored by varying the cylinder radii and the packing density (specified by *p*). The cylinder radii were chosen to yield a useful range of EAS cross-sectional areas (EAS areas) under the abutting case. That is, the cylinder radii were chosen such that abutting EAS areas had specific target radii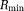. The value of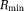 reflects the EAS area under the tightest possible packing.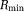 is related to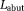 for square and hexagonally cylinders as

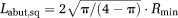
13

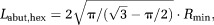
14respectively. All combinations of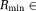 {0.05, 0.1, 0.5, 1, 5} µm and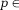 {1.00, 1.03, 1.06, 1.09, 1.12, 1.25, 1.50, 2.00} were simulated.

For randomly packed cylinders, the cylinder radii were chosen to have a gamma distribution corresponding to the genu of the human corpus callosum 41 as well as gamma distributions with twice and thrice the cylinder radii. Simulations were performed with cylinder volume fractions of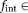 {0.73, 0.6, 0.5, 0.4, 0.3} by varying the cylinder spacing while keeping the cylinder distributions constant (Fig. 4e—more detailed versions can be found in Fig. S1 of the Supporting Information). In total, the diffusion spectra for 15 randomly packed geometries (three distributions at each of the five volume fractions) were simulated. Each simulation consisted of 500 cylinders in a base voxel, which was then tiled. Particles diffusing across any given edge of a tile reappear at the opposite edge, which enables an efficient and accurate simulation of a large-scale geometry based on a smaller-scale representation.

Diffusion gradient frequencies were chosen to simulate as much of the dynamic range of the diffusion spectra as possible with the available computational power. Apodized cosine oscillating gradients 17 were used to minimize the encoding spectrum side lobes and were simulated at frequencies of {2, 4,…, 10, 20,…, 100, 200,…, 1,000, 2,000,…, 10,000, 20,000,…, 100,000, 200,000,…, 1,000,000} Hz. The duration of the oscillating gradient waveforms was chosen to sample narrowly about the target frequency, with a sampling width of 500 ms (2 Hz FWHM) for frequencies below 10,000 Hz and 4 ms (250 Hz) for higher frequencies. The gradients were applied perpendicular to the cylinder axes. The peak amplitude of each waveform was chosen to produce a *b* value of 1 ms/µm^2^.

The other simulation parameters are summarized in Table2. A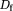 of 2 µm^2^/ms was used and no noise was added. Simulations were run in parallel on a computing cluster of Intel Xeon X5647 processors. The run times for simulations of 10,000 walkers each were approximately 12, 60, and 120 h of processor time for 250,000, 2,000,000, and 8,000,000 time steps, respectively.

**Table 2:** Monte Carlo Simulation Parameters

Gradient			
Frequency (Hz)	Duration (ms)	Number of time steps	Walkers
2–10,000	500	8,000,000 ( 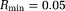 µm),	10,000 ( 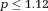 ),
			30,000 ( 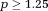 ),
		2,000,000 ( 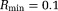 µm),	100,000 (random packing)
		250,000 ( 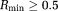 µm and random packing)	
5,000–1,000,000	4	400,000	10,000 ( 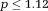 ),
			30,000 ( 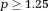 ),
			100,000 (random packing)

In the first frequency range, more time steps were required for small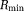 (periodic packing only) to keep the walker displacement per step much less than the distance between cylinders. Otherwise,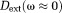 would be underestimated. In the second frequency range, the gradient duration was reduced due to computer memory constraints.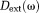 was comparable where the frequency ranges overlapped. The number of walkers was increased for large *p* (periodic packing only) to compensate for the decreased signal of the restricted component.

The signal attenuation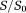 at each frequency was converted to a corresponding point in the diffusion spectrum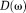 via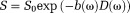 (where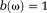 ms/µm^2^ in our simulations). Particles within impermeable, uniform cylinders (intra-axonal) were also simulated to verify that the simulated diffusion spectrum matched the known expression 12.

### Forward Model Comparison to Simulated Data

The primary goal of this article is to compare the simulated diffusion spectra to the forward model using the known properties of the simulated microstructure. EAS diffusion spectra are calculated using the model for each of the square and hexagonal packing simulated cases. In these cases of regular packing, the ground truth can be calculated straightforwardly and compared to the simulated diffusion spectra. Forward modeling was not performed for randomly packed cylinders as physical interpretations for *R*_0_ and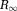 could not be found.

### Model Fitting

Fitting for the model parameters from the simulated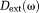 data was used to determine whether parameter values can uniquely describe the geometries considered. Bayesian fitting with the Metropolis–Hastings algorithm was performed, with the fit initialized by the result of nonlinear least squares. For the nonlinear least squares analysis, the initial value of lambda was given by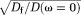 for all cases; the initial values of *R*_0_ and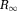 were arbitrarily set to 10 and 1 nm, respectively, for regular packing; and the initial values of the *R*_0_ mean, *R*_0_ standard deviation, and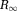 were 1 µm, 10 nm, and 500 nm, respectively, for random packing.

For regular packing, the simulated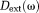 curves were fitted for *λ*, *R*_0_, and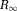. These three parameters were also calculated from analytical expressions for *λ*,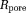, *S*/*V*, and *p* and compared with the fitted values to ascertain whether there is a one-to-one correspondence, which is required for the model to unambiguously describe the microstructure. For random packing, the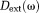 curves were fitted for *λ*, the mean and variance of a gamma distribution used to describe *R*_0_, and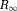.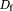 was assumed to be that used in the simulations.

### Describing the Microstructure

The fitted model parameters were used to calculate values that describe the microstructural environment of the EAS. For square and hexagonal packing, *λ* was calculated numerically from *p* using existing expressions 34,35. Then,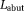 and *L* were calculated using Eqs. 10 and 11 in combination with the expressions for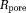 and *S*/*V* in Appendix B.

While analytical calculation of pore sizes can be performed for regular packings, this is not generally possible for random packings. Instead, the EAS of the simulated randomly packed cylinders was segmented into extracylinder pores using a custom algorithm. The resulting distribution for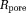 was compared to the mean value of the fitted *R*_0_ distribution.

All segmentation and fitting were performed in MATLAB (R2011b, The MathWorks, Natick, MA).

## RESULTS

We begin by assessing the overall ability of the model to predict simulated diffusion spectra across a wide range of square and hexagonal cylinder packing geometries, including packing densities and spatial scales. We then assess the uniqueness of the model parameters *λ*, *R*_0_, and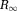 when fitted to diffusion spectra under ideal (noiseless) conditions. Finally, we compare the fitted parameters to physically meaningful geometrical values (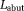 and *L* for square and hexagonal packing and the mean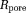 for random packing).

### Forward Model Comparison to Simulated Data for Regular Packing

**Figure 5:**
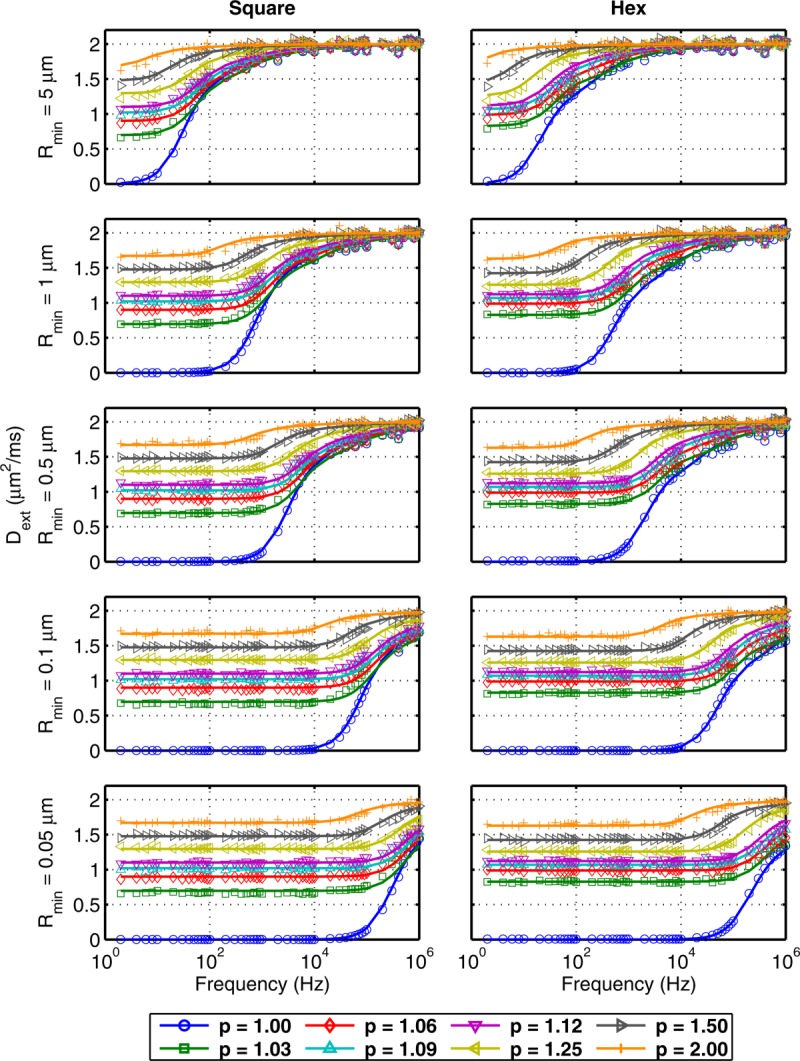
Simulated (markers) and predicted (–) EAS diffusion spectra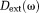 for square and hexagonally packed cylinders.

Simulated and predicted EAS spectra are shown in Figure 5 for square and hexagonal packing. The simulated and modeled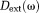 match well over a broad range of geometries. The quality of model agreement can be best appreciated by examining the residuals (data subtracted from model) found in Figure S2 of the Supporting Information. Overall, the residuals are very small, although there is a negative deviation from zero for square packed cylinders with relatively tight packing (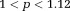). This deviation occurs near the frequency of the initial rise in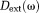, determined by the parameter *R*_0_. It is worth noting that the residuals in these regions of the spectrum are particularly sensitive to differences between the simulations and predictions since this is where the spectrum is changing most rapidly. Put another way, the residuals measure the vertical difference between model and simulation, which can be large in regions of rapid change, even for curves that by eye are only subtly different. Hexagonal packings do not show this feature in the residuals, indicating a better overall model prediction (i.e., residuals that are generally very close to zero).

Simulated and predicted EAS spectra are shown in Figure 7 for random packing. The simulated and modeled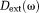 also match well over a broad range of geometries. The residuals are shown in Figure S3 of the Supporting Information and have smaller absolute values than those for regular packing.

### Model Fitting

**Figure 6:**
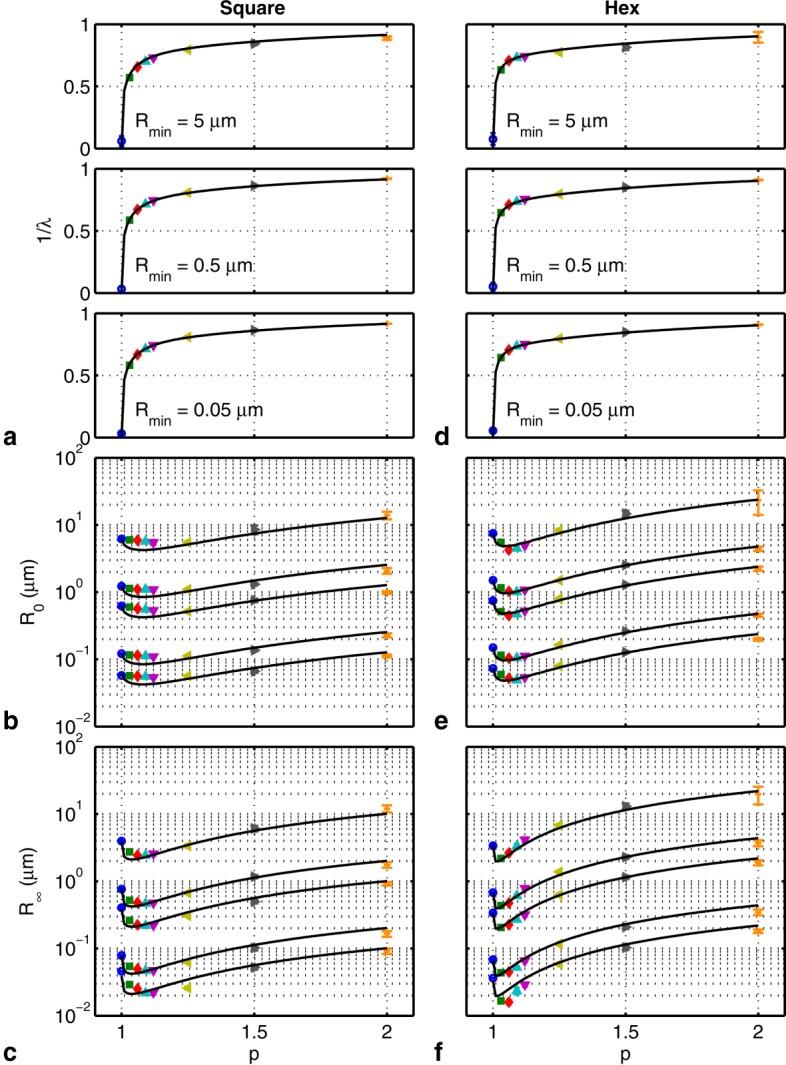
Fitted (mean ± SD; markers) and predicted (–) model parameters (a)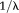 (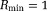 and 0.1 µm are similar and not shown), (b) *R*_0_, and (c)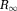 for square packed cylinders. The curves in b and c denote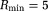, 1, 0.5, 0.1, 0.05 µm (top to bottom). d, e, f: As a, b, and c, but for hexagonally packed cylinders.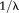 is plotted instead of *λ* to show the abutting case (*p* = 1). The markers are as in Figure 5.

The tortuosity parameter is straightforward to interpret and is compared to analytically calculated values for square and hexagonally packed cylinders in Figure 6a,d, respectively. The agreement between the fitted and theoretical values is very good in all cases.

**Figure 7:**
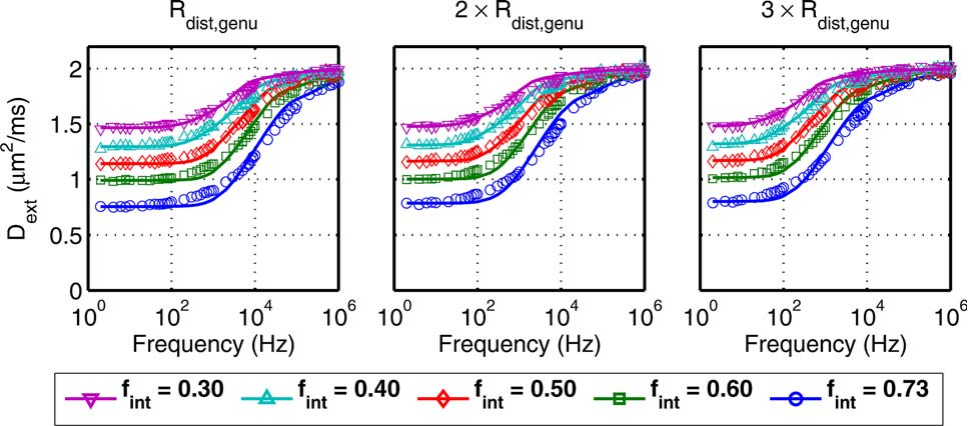
Simulated (markers) and predicted (–) EAS diffusion spectra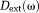 for randomly packed cylinders over a range of five cylinder volume fractions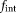 and cylinders with radius distributions that are one, two, and three times those of axons in the genu (left to right).

The abstract model parameters *R*_0_ and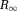 are crucial to evaluate because they only have an empirical relationship to parameters of the simulated geometries. Thus, it is important to establish that these parameters (a) capture the expected relationships with *λ*,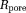, *S*/*V*, and *p* as indicated in Eqs. [10]–[12] and (b) exhibit an (approximately) one-to-one mapping with microstructural geometry. If there were no unique mapping, the estimation of the microstructural geometry would be confounded. Model fits to simulated data show that the radius parameters are in reasonable agreement with calculated values for square and hexagonal packing as shown in Figure 6b,c,e, and f. For square packing, the fitted values of *R*_0_ overestimate the expected *R*_0_ for small values of *p*, although the general trends are in good agreement. One possible cause of disagreement for square packing are larger displacements for a given diffusion time due to the presence of straight paths between cylinders, which are not accounted for in the calculation of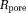. The fitted values of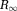 generally match the model well. The parameters *R*_0_ and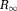 appear to give a unique mapping to all the combinations of EAS length scales and packing densities simulated.

For square and hexagonal packing, the calculated values for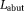 and *L* from *R*_0_ and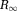 versus true values are presented in Figure S4 of the Supporting Information. The estimates match the simulated values over a wide range of spatial scales. The accuracy is high despite the complicated geometry of the EAS, which effectively combines a variety of spatial scales.

**Figure 8:**
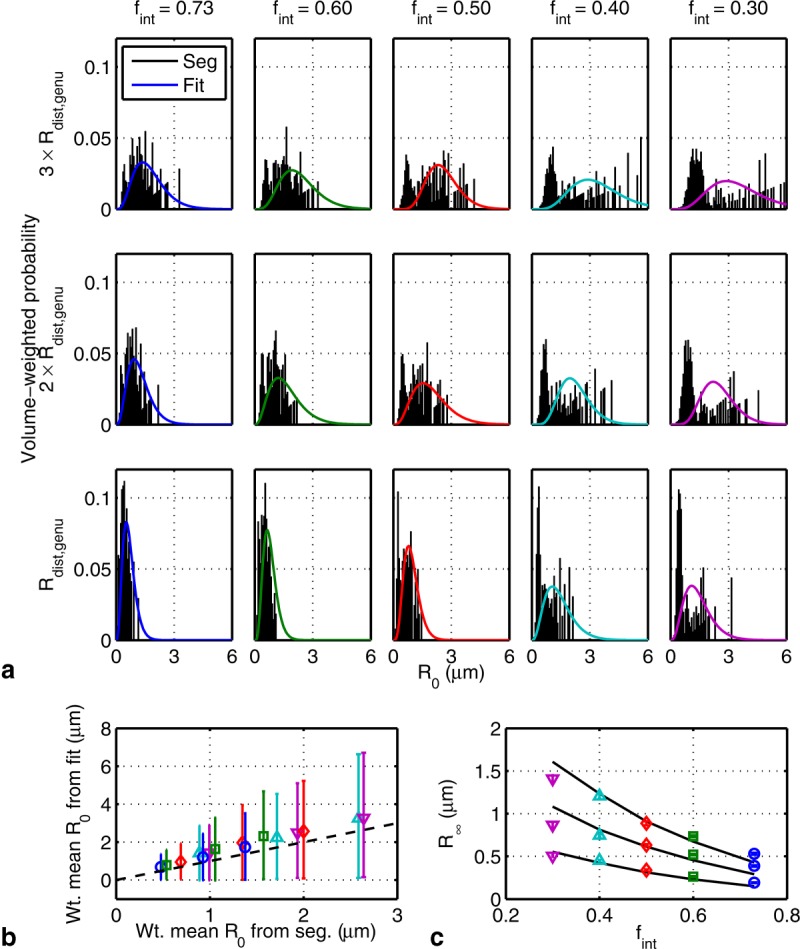
a: Distribution from segmentation (bars) and prediction (–) for model parameter *R*_0_ weighted by cylinder volume fraction. b: The mean and standard deviations of the volume-weighted *R*_0_ distributions from fitting versus segmentation corresponding to those in a. c: Fitted (mean ± SD; markers) and predicted (–) model parameter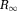 for randomly packed cylinders. The curves denote radius distributions that are three, two, and one times those of axons in the genu (top to bottom). The markers are as in Figure 7.

For randomly packed cylinders, the model using a distribution of *R*_0_ fits well to the diffusion spectra (Fig. 7; residuals, which are smaller than those for the regular packing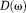 are shown in Figure S3 of the Supporting Information). The single *R*_0_ model used for regular packing did not fit the random packing data well (not shown), which indicates the need for an *R*_0_ distribution. The *R*_0_ distributions from fitting and from the EAS segmentation algorithm, when weighted by the volume of each pore, are shown in Figure 8a. At high packing densities (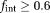), they broadly match, but at low densities, a bimodal distribution is evident. The random placement of cylinders by Camino is likely the cause of the bimodality. At low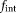, the cylinders exhibit clumping from random placement (see Figure S1 in the Supporting Information) and would yield a bimodal distribution. An alternative arrangement of cylinders with the same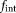, but more even spacing, should yield a more unimodal distribution. At high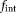, Camino spaces the cylinders more evenly to minimize the amount of EAS, which would automatically yield a unimodal distribution. Without volume weighting, there is an even stronger bimodal distribution at all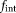, which suggests the need for volume weighting. The means of the distributions from fitting and segmentation closely match (Fig. 8b). The fitted values of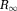 match the model quite well (Fig. 8c).

## DISCUSSIONS

In this article, we present an analytical model for the diffusion spectrum of the EAS that is applicable to square, hexagonally, and randomly packed cylinders. This is an idealized view as axons are neither perfectly cylindrical nor impermeable and glia are ignored. However, this kind of simulation, as provided by Camino, has nevertheless been very powerful for gaining insights into diffusion in white matter. Proper modeling of the EAS diffusion spectrum will aid in the correct interpretation of oscillating gradient diffusion measurements. Although the diffusion spectrum of the EAS is known not to be spectrally flat 27,28, we believe that our empirical model is the first to predict the EAS diffusion spectrum across the entire frequency range with parameters that have a one-to-one correspondence to the microstructure of idealized cylinders. Below we discuss alternative models and considerations when acquiring and analyzing real data.

### Alternative Models

Our model is the simplest we could devise that would match all of the simulated data. While a model that describes EAS diffusion with, say, one free and two restricted components with fixed radius across frequencies would also predict the data, that would entail four parameters (e.g., tortuosity, volume fraction of one restricted component, and the radius of each) instead of three with the model presented in this article, while not giving improved accuracy (data not shown).

Alternative formulations for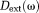 have been proposed. The closest to the one presented in this article is that of Lasič et al. 42. Their model also uses Eq. 4, but for the purpose of characterizing the exchange of water molecules between microscopic water droplets. The restricted diffusion attenuation is modeled as a sum of diffusion spectra weighted by droplet volume and size distribution as well (cf. Eq. 8). However, in their model, the pore size does not vary with frequency. Furthermore, the motivation for our model differs in that we seek to describe the microstructure of the EAS.

Parsons et al. 28 found a relationship between the estimated pore size around tightly packed beads and the bead size from a model similar to that described by Eq. 4. However, the restricted component of their model, like that of Lasič et al., also uses a constant pore size over all frequencies, which describes the intermediate frequency regions of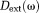 well, but results in less accuracy at high frequencies. Our model describes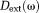 with good accuracy at all frequencies and applies to tight as well as sparse packing.

Complementary to the frequency domain diffusion spectrum models above, there exists a large body of literature that considers the diffusion coefficient in the time domain as a function of diffusion time *τ* measured with pulsed gradients. Latour et al. 43,44 used a Padé approximant to interpolate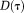 and extract tortuosity and pore size. Our model empirically models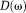 to estimate similar parameters. There is also much work on the calculation of tortuosity for many geometries [in two dimensions with cylinders 45,46 and random shapes 47 and in three dimensions 48–52] and relating it to microstructure. Our work, which aims instead to relate the whole of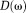 to microstructure, can provide more information than tortuosity would alone.

Alternate pulse sequences have also been proposed for increased sensitivity to axon diameter. Oscillating gradient sequences with variable waveform periods 53,54 and time-varying direction 55 have been proposed. As well, numerically optimized pulsed gradient sequences have also been proposed 56,57. However, measurement of the extremely small length scales of the EAS will pose significant challenges.

### Practical Considerations

There are practical considerations to be noted when attempting to measure the diffusion spectrum [typically with an oscillating gradient spin echo sequence 58] including the *T*_2_ signal decay, hardware constraints, and peripheral nerve stimulation of the subject. Measurements at low frequencies are governed by *T*_2_ decay, which limits the gradient waveform duration and thus the minimum frequency. Measurements at high frequencies are limited by the diffusion contrast achievable and by peripheral nerve stimulation. Sufficient diffusion contrast requires a large number of gradient oscillations (also limited by *T*_2_ decay) and/or high gradient amplitude (limited by hardware). Peripheral nerve stimulation could also be problematic at higher frequencies, which require high gradient slew rates. This could potentially be alleviated by the use of head-only gradient coils, which can achieve higher slew rates before the onset of peripheral nerve stimulation 59,60.

The table in the Supporting Information lists typical maximum frequencies for various types of MR systems and shows that current clinical systems are insufficient for probing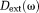 of white matter (cf. Fig. 7). Animal systems can probe the frequencies at which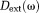 begins to change and microscopy systems can access a significant portion.

### Model Considerations

The model presented in this article is a highly idealized picture of the EAS, and several factors that may contribute to measurements of the diffusion spectrum of white matter have been neglected. For instance, measurements will also have contribution from the intra-axonal space, and separating the two compartments using diffusion spectrum measurements is challenging. Other complications include nonparallel and/or noncylindrical axons, exchange between different compartments, and the presence of other cellular processes, such as glial cells, which may also contribute to the measured shape of the diffusion spectrum.

In order for our model to be deployed in real tissue, it will be necessary to account for both intra- and extracellular spaces. The present work focused solely on the extracellular space as an important tissue compartment that has received limited attention in terms of its diffusion spectral characteristics. A future challenge will be to build a single model with both compartments. Assuming no exchange, forward modeling should be straightforward (a volume-weighted summation of signals). Model fitting, however, will require a careful parameterization to link, for example, intracellular radii and volume fraction to the geometric properties of the extracellular space. This nontrivial problem is the topic of ongoing research in our group.

Although our interest in white matter led us to simulate cylinders on the scale of axons, technological limitations prevent the measurement of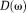 at sufficiently high frequencies. Information about the size of the extracellular space is primarily reflected in the transition region of the diffusion spectrum, meaning that accurate estimation of compartment sizes requires sampling at frequencies up to the maximum gradient in the transition. Assuming an extracellular radius of 0.1 µm, frequencies of approximately 60 kHz (see Fig. 7) will be required to measure the diffusion spectrum for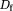 = 2 µm^2^/ms (in vivo) and 6 kHz for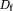 = 0.2 µm^2^/ms (fixed tissue, calculation not shown). The larger length scale of the rat sciatic nerve (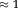 µm axon and pore radii) potentially allows estimation of parameters using animal scanners with a maximum gradient amplitude of 750 mT/m (allowing a *b* value of 0.6 ms/µm^2^) at frequencies up to 300 Hz. This could be useful for tracking the progression of experimental autoimmune neuritis 61 and diabetic nerve regeneration 62. Moreover, there may be other cellular microstructures of interest outside of the nervous system characterized by a sufficiently large length scale for this model to be of interest.

## CONCLUSIONS

We have presented an empirical model for the diffusion spectra of particles in the EAS. This model was able to predict the spectra from extensive Monte Carlo simulations. We demonstrated the estimation of tortuosity, pore size, and surface-to-volume ratio for parallel cylinders with a variety of packing geometries and a wide range of spatial separations. This model can potentially be used to estimate the geometric properties of cylindrical structures such as those found in white matter.

## Appendix A: Diffusion spectrum for a cylinder

The diffusion spectrum for particles within an impermeable cylinder is given by 12

15

where

16

17

and are the roots of.

## Appendix B: Geometric quantities for periodically packed cylinders

### Effective Pore Radius

The effective pore radius is defined to be the mean distance from the pore centroid to boundary in a plane perpendicular to the cylinders. For square packed cylinders, it is given by

18

and, for hexagonally packed cylinders,

19

where represents the angle blocked by a cylinder with respect to the pore center.

### Surface-to-Volume Ratio

For square packed cylinders, the ratio of surface area to pore volume *S*/*V* is given by

20

and, for hexagonally packed cylinders,

21
