# Supplementary material for: A model for extra-axonal diffusion spectra with frequency-dependent restriction
Source: Magn Reson Med. 2014 Jul 15;73(6):2306–20. doi: 10.1002/mrm.25363 (PMC4682484; doi:10.1002/mrm.25363)
Supplement: Supplementary file 1 — Supplementary Information [file mrm0073-2306-sd1.pdf]

Supporting Information

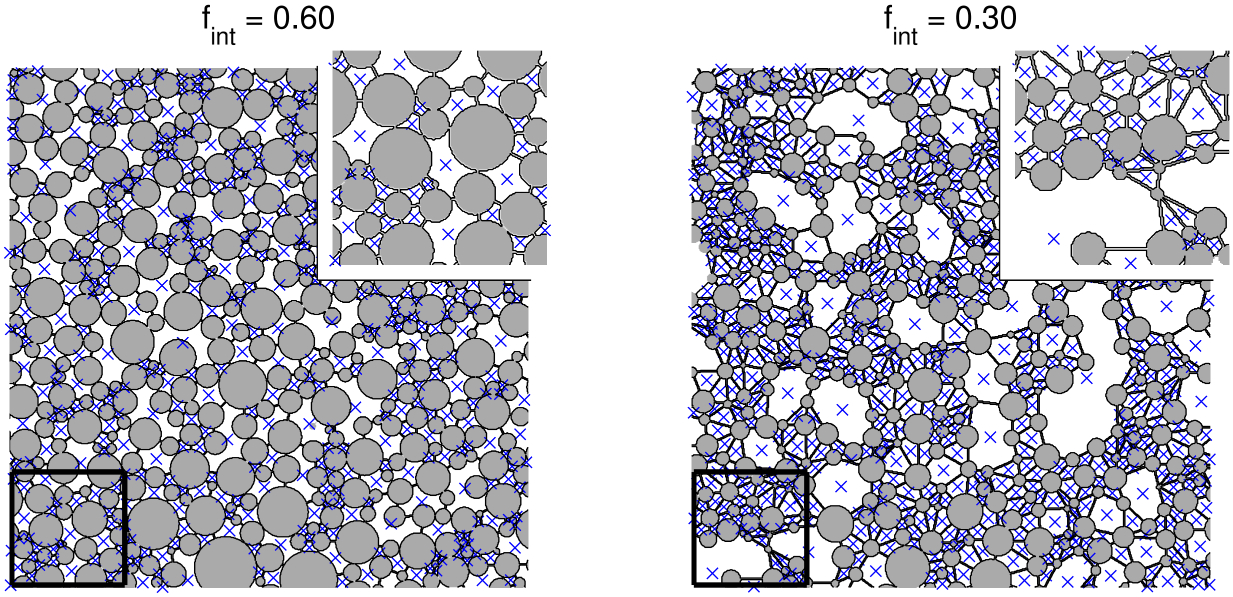

Figure S1: EAS pore segmentation for randomly packed cylinders with gamma-distributed radii for cylinders volume fractions $f_{\mathrm{int}} = 0.60$ and 0.30. Pore centroids are indicated by $\times$. Inset are magnified views of the boxed regions.

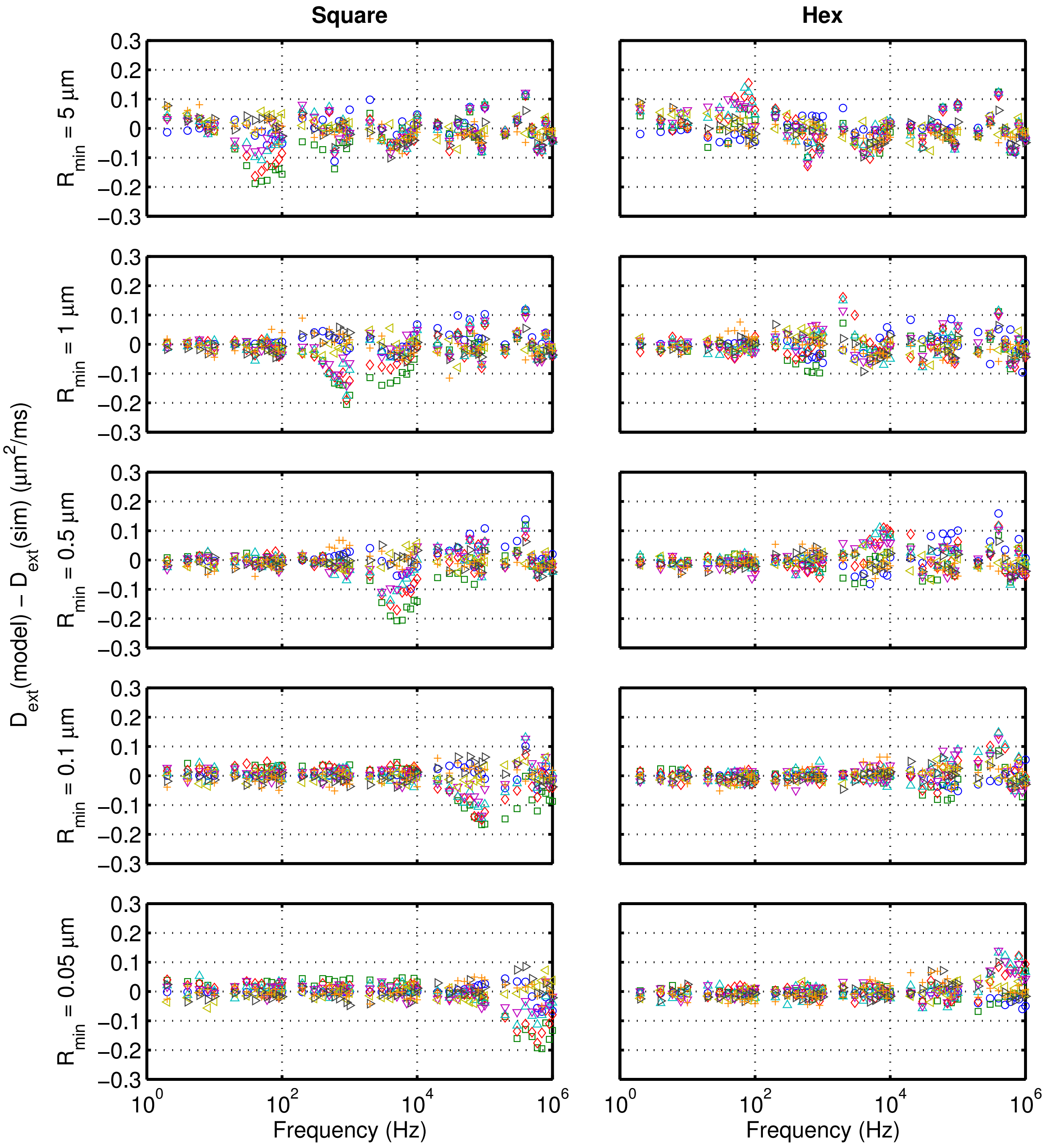

Figure S2: Residuals between simulated and predicted EAS diffusion spectra $D_{\mathrm{ext}}(\omega)$ for square and hexagonally packed cylinders. The markers are as in Fig. 5.

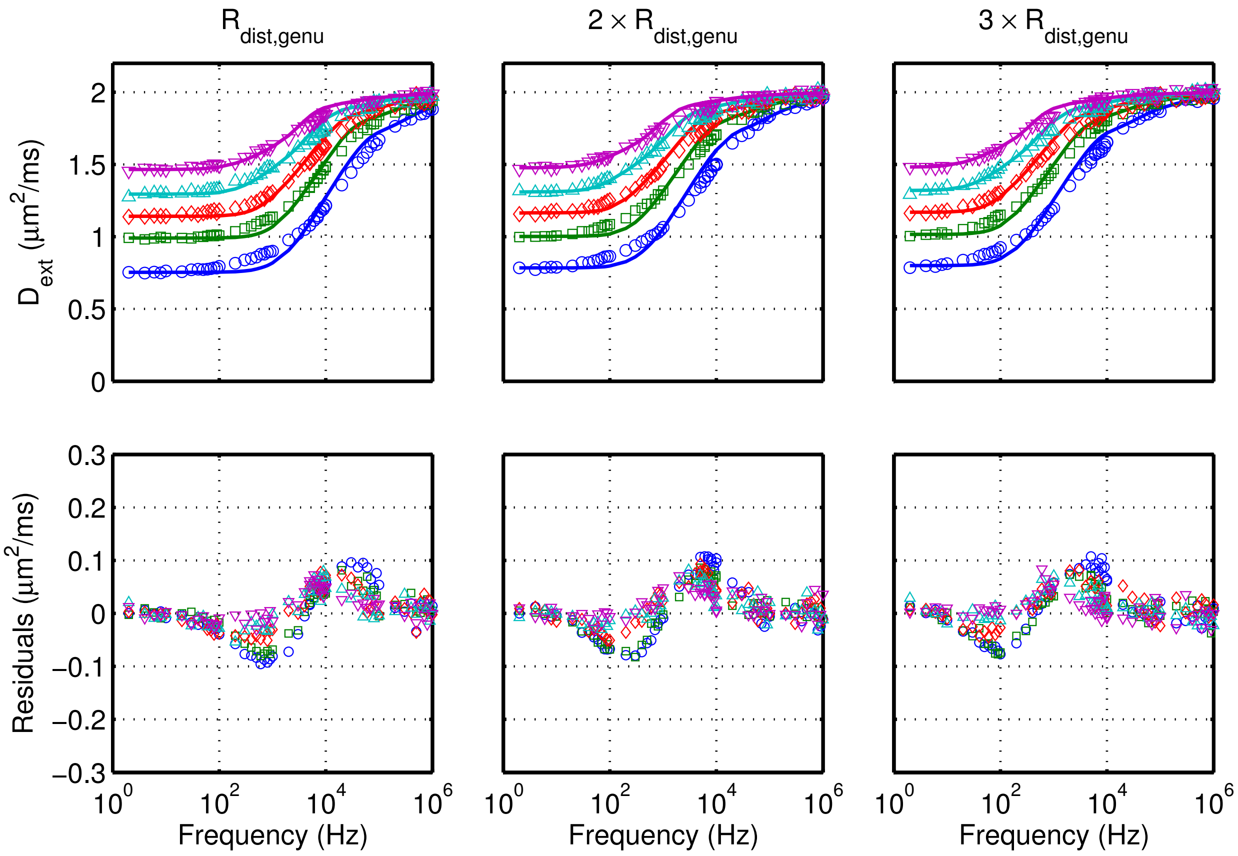

Figure S3: Simulated and predicted EAS diffusion spectra $D_{\text{ext}}(\omega)$ for randomly packed cylinders and their residuals. The markers are as in Fig. 7.

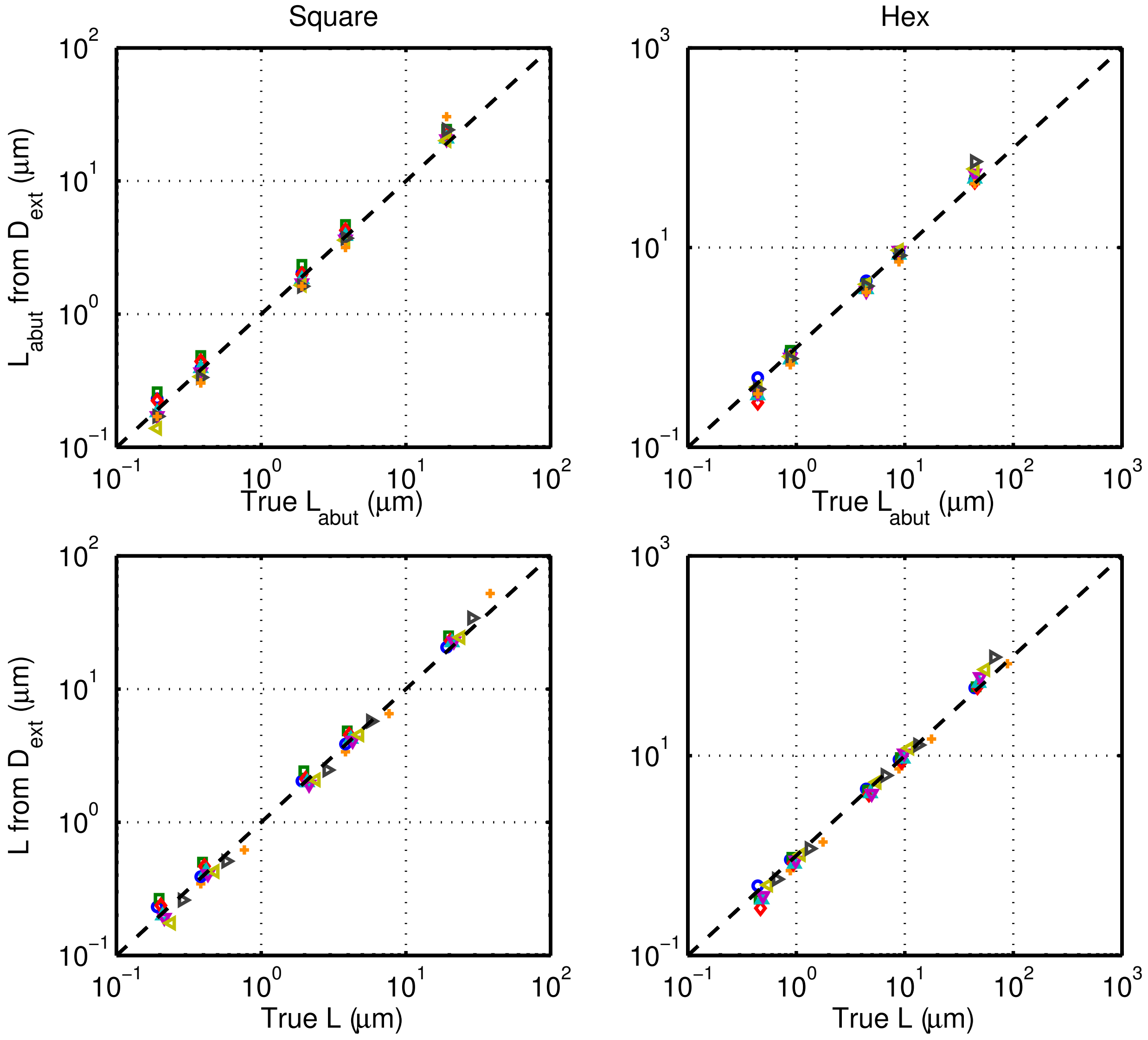

Figure S4: Abutting cylinder separation L_{abut} and cylinder separation L for square and hexagonally packed cylinders calculated from the fitted values of λ, R_0, and R_∞. The markers are as in Fig. 5.

Table S1: Recent oscillating gradient experiments employed in literature

MR system	Max. frequency (Hz)	b (ms/μm^2)	Ref.
Human	50	0.3	(20)
Human	63	0.2	(19)
Animal	150	0.7	(22)
Animal	250	0.4	Xu et al. PLoS ONE 2012;7:e41714.
Microscopy	1,000	3.2	(21)
